# Antibacterial and nonlinear dynamical analysis of flower and hexagon-shaped ZnO microstructures

**DOI:** 10.1038/s41598-020-59534-x

**Published:** 2020-02-13

**Authors:** Rajat K. Saha, Mrinal K. Debanath, Bishaldip Paul, Subhash Medhi, Eeshankur Saikia

**Affiliations:** 1 Department of Applied Sciences, Gauhati University, Guwahati, 781014 India; 2 Department of Bioengineering and Technology, Gauhati University, Guwahati, 781014 India

**Keywords:** Structural biology, Structural properties

## Abstract

The present study reports the antibacterial properties of flower-shaped ZnO (FZnO) microstructures and its comparison with that of hexagon-shaped bulk ZnO (BZnO) nanostructures. The samples are prepared successfully by wet chemical method and the surface morphologies, structures and size of the ZnO samples are characterized by X-ray diffraction (XRD), Scanning Electron Microscopy (SEM), Transmission Electron Microscopy (TEM), BET adsorption isotherm, and Photoluminescence (PL) Spectroscopy. The SEM and TEM images of the sample have confirmed flower-shaped structure of the ZnO. The materials are also analyzed by using an innovative tool called Lacunarity, a nonlinear dynamical (NLD) tool for proper understanding of the inherent surface properties of the particles formed, comparing the results estimated with the BET results obtained, thereby confirming our proposition to use it as an important parameter in predictive models. In this new approach, geometry of the surface structure is being associated with biological properties, in order to come up with easier ways to identify materials for any such applications where rich surface structure is desired. The photocatalytic activity of the flower-shaped material is carried out to find out its optical properties as another marker for confirming the antimicrobial activities. It has been reported for the first time that the prominent antibacterial activities are favoured by the FZnO microstructure having lesser Lacunarity, significantly better than its bulk counterpart, for inhibiting gram negative - *Escherichia coli* microorganism.

## Introduction

Nanotechnology, at present, pertains to creation of useful materials, devices, and systems through appropriate manipulation of matter. A significant number of researchers have reported that formation of ZnO nanostructures in different shapes, such as, nanorod, nanoplates, nanowire^1^, flower-shaped ZnO microstructures^2^, etc. In the present work, two important attributes, capping and molarity, are used to control the particle size and their agglomeration^3^. It is reported in W Yu *et al*. (2016), that photo-generated electrons in ZnO are much lighter than their corresponding holes, which indicates that it belongs to the n-type semiconductor^4^. This result paves the way for obtaining excitation energy of electrons in ZnO that determines its optical property. ZnO materials are good inhibitors of microorganisms, and it is believed that this activity is enhanced in case of ZnO due to its high surface morphology. The capped ZnO usually shows up higher antibacterial activity than the uncapped one^5^. In the present work, flower-shaped ZnO (FZnO) microstructures in PVP matrix and uncapped hexagon-shaped bulk ZnO (BZnO) nanostructures are synthesized for the study of their antimicrobial properties. The as-prepared samples are characterized by X-ray diffraction (XRD)^6^, Scanning Electron Microscopy (SEM) and Transmission Electron Microscopy (TEM) to find their size and shape^7,8^. Further, the FZnO are characterized by UV-spectra, Photoluminescence (PL) Spectra for finding its band gap and optical properties for biological applications. The average granule sizes are also calculated by using Debye Scherrer method^4^ and they are verified further with those obtained from TEM images. The inherent nonlinearity involved in the process of formation of the ZnO nanoparticles (NPs) determines the factors that are thought to be responsible for enhanced inhibition of microorganisms^5^. The richness in surface properties is also one of the factors for high inhibition of microorganisms. Therefore, the samples are further characterized for finding out their effective surface area, porosity and adsorption using surface area analyzer. The pore size distribution of the samples are carried out for finding their surface properties. Also an attempt is made for quantifying the nonlinearity in the surface structures of the ZnO, using one of the tools of Nonlinear Dynamics (NLD), called Lacunarity Analysis^9,10^. The optical properties of ZnO NPs paved the way for determining their antibacterial nature, and hence it showed good results in inhibiting pathogens^11^. The photocatalytic degradation & enhancement in the antibacterial activity against the pathogen *Escherichia coli* (*E. coli*) are believed to be caused due to increase in surface area of ZnO NPs, suggesting that both the properties are governed by similar mechanisms^12^. It was also reported that the optical property of FZnO microstructure and its antimicrobial activity were regulated by surface-volume ratio of the granules^11^. Semiconductor photocatalysts have captured considerable attention because of their ability to utilize solar energy, for the degradation of organic pollutants^13^. It was also reported that ZnO NPs exhibit potential as an efficient photocatalyst in case of the Methylene Orange dye, and had proved to be an effective antibacterial agent for elimination of bacteria from the contaminated water. The optical property of as-prepared sample FZnO is analyzed by carrying out photocatalytic activity for exploring the possibility of biological applications. Finally, an attempt is also made to carry out analysis of the antibacterial activity of the as-prepared sample on gram-negative microorganisms *E. coli*.

## Experimental Details

### Materials

The materials were purchased from the commercial market. Zinc nitrate hexahydrate [Zn(NO_3_)_2_.6H_2_O] and ammonium hydroxide (NH_4_OH) as the base materials and de-ionised distilled water as dispersing solvent were purchased from Merck Specialities Private Limited, Mumbai, India. The capping agent Polyvinylpyrrolidone (PVP) were purchased from LOBA Cheme, Mumbai, India. The above materials were used to prepare the FZnO and BZnO nanoparticles. Filter paper bearing number Cat No 1542-125 were purchased from Whatman, UK and the glassware used for the purpose were purchased from Borosil Glass Works Limited, Mumbai, INDIA. The culture media were bought from HiMedia Pvt. Ltd., Mumbai, India.

### Synthesis

The syntheses of FZnO and BZnO have been carried out by simple wet chemical method^14,15^. Both the samples fabricated are undoped, so as to avoid the new phase formation of the compound and in the increase in their lattice parameters. Firstly, for FZnO 0.075 mol solution (soln) of Zn(NO_3_)_2_.6H_2_O is used as a zinc source. 100 ml of 0.075 mol soln of Zn(NO_3_)_2_.6H_2_O is stirred constantly for 30 mins at 60 °C (soln A). Subsequently 3 wt% of PVP is stirred constantly at 60 °C for 30 mins (soln B). Secondly, NH_4_OH was slowly added drop by drop into the soln A and was stirred at room temperature for 15 mins and simultaneously the pH of the soln was measured. It is observed that a white precipitation (soln C) is formed when the pH of the soln attains 7.5 and thereafter no more NH_4_OH is added. However, nanoparticles tend to aggregate because of their high surface-to-volume ratio and high surface energy. The capping and change in molarity along with pH of the soln, played an important role in determining the final morphology of the microstructures^16–18^. The mixture of soln B and soln C is stirred continuously for 1 hour at 60 °C and allowed to return gradually to room temperature. Then, the whole soln of ZnO is settled down for 24 hours in a dark chamber. Lastly, the precipitate is filtered and washed with distilled water for dissolving the impurities present if any, and is dried at 60 °C in oven for 10 hours^14^. Secondly, for synthesizing BZnO, the same procedure is adopted except that 0.1 mol soln of Zn(NO_3_)_2_.6H_2_O and no capping agents were used.

The mechanisms for ZnO crystal growth are shown below. The chemical change of Zn(OH)_4_^2−^ or Zn(NH_3_)_4_^2+^ are according to the forerunner reactions^3,14^:

$${\rm{Zn}}{({{\rm{NO}}}_{3})}_{2}.6{{\rm{H}}}_{2}{\rm{O}}\leftrightarrow {{\rm{Zn}}}^{2+}+2{{\rm{NO}}}_{3}^{2-}+6{{\rm{H}}}_{2}{\rm{O}}$$

$${{\rm{NH}}}_{4}{\rm{OH}}\leftrightarrow {{{\rm{NH}}}_{4}}^{+}+{{\rm{OH}}}^{-}\leftrightarrow {{\rm{NH}}}_{3}+{{\rm{H}}}_{2}{\rm{O}}$$

$${{\rm{Zn}}}^{2+}+4{{\rm{NH}}}_{3}\leftrightarrow {\rm{Zn}}{({{\rm{NH}}}_{3})}_{4}^{2+}$$

$${\rm{Zn}}{({{\rm{NH}}}_{3})}_{4}^{2+}+2{{\rm{OH}}}^{-}\leftrightarrow {\rm{ZnO}}+4{{\rm{NH}}}_{3}+{{\rm{H}}}_{2}{\rm{O}}$$

$${{\rm{Zn}}}^{2+}+4{{\rm{OH}}}^{-}\leftrightarrow {\rm{Zn}}{({\rm{OH}})}_{4}^{2-}$$

$${\rm{Zn}}{({\rm{OH}})}_{4}^{2-}\leftrightarrow {\rm{ZnO}}+{{\rm{H}}}_{2}{\rm{O}}+2{{\rm{OH}}}^{-}$$

The growth of ZnO were based on the pH of the soln and solvent used as well, which enhanced the formation of ZnO structure. The factors also influenced the size and shape of the fabricated nanoparticles^2,3,14^. At low reaction temperature (60 °C) on account of NH_3_ and Zn(NH_3_)_4_^2+^, larger Zn^2+^ are converted in the function of OH^−^. As a result, more ZnO NPs aggregate spontaneously along active sites for the growth of flower-shaped ZnO microstructure due to their high surface-to-volume ratio and high surface energy. The surface pattern of ZnO structures obtained from Zn(OH)_4_^2−^ controls the extension of various crystal facets that leads to the evolution of anisotropic structures, such as, flower-shaped ZnO^14,19^. The hexagonal ZnO has a positive polar plane Zn (0 0 0 1) rich in Zn, six symmetric non-polar {10–10} planes of the side facets, and a negative polar plane (000–1) rich in O. It is found that different planes have different growth rates as V_(000–1)_ < V_(10–10)_ < V_(10–11)_ < V_(0001)_. The negatively charged complex ions Zn(OH)_4_^2−^ are preferentially adsorbed onto the positively charged Zn (0 0 0 1) faces followed by dehydration so as to move into the crystal lattice along (0 0 0 1) faces favoring the preferential development and results into the flower-like ZnO.

Besides, capping agent PVP tends to adsorb on the {10–10} planes of the six symmetric side facets of the growth nuclei, thereby it allows to occur only along the polar axis^14,20,21^.

## Methods

### Characterization methods

Powder X-ray diffraction (XRD) pattern of the fabricated BZnO and FZnO nanoparticles are recorded by Philips X-ray Diffractrometer (X’ Pert Pro) with *Cu K*_*α1*_ radiation (*λ* = 1.5406 *Å*). Field Effect Scanning Electron Microscope (MIRA3 TESCAN) and Transmission Electron Microscope (JEM-2100, Jeol) were used for confirmation of FZnO microstructures^20^. The optical absorption spectra of ZnO dispersed in methylene blue (MB) dye are recorded by a UV–vis spectrophotometer (HITACHI U - 3210). The Photoluminescence spectra of the FZnO were measured at room temperature using Fluorescence Spectrometer (HITACHI–2500). The BET surface area of the synthesized BZnO and FZnO samples were characterized using Tristar 3000 V6.08 A Brunauer Emmet Teller Surface Area Analyzer.

### Photo luminescence (PL) spectroscopy analysis

The Photoluminescence (PL) technique is a simple characterization method for determining the optical properties and electronic structure of materials. A small amount of the sample BZnO was added to get dispersed into de-ionized distilled water. For analyzing the as-prepared sample, the nanostructured powder solution was then transferred into a 10 mm sampling quartz cuvette for the experiment to carry out, and the data and related spectrum obtained are then recorded. Similar procedure was adopted for FZnO^14^. Through the analyses, the band structure of the materials of the as-prepared samples are studied for finding their band gaps and other optical properties, and also for finding out interstitial defects of the materials^21^.

### BET isotherm analysis

Nitrogen adsorption measurements were made for the Micromeritics of the samples to obtain their effective surface area, pore size distribution and pore structure properties. The Brunauer Emmett Teller (BET) surface area and adsorption energies were calculated from the adsorption isotherm data using absorptive molecular nitrogen gas. The BET isotherm analysis is a widely-used and well-behaved method for extracting effective surface areas and adsorption energies from isotherm data. The method is based on a model of multilayer adsorption which is based on the adsorption occurs on adsorbing sites and on top of adsorbed molecules, that the number of adsorbing sites per layer is constant. The plot between the adsorbed energy and relative pressure is obtained from the fundamental straight line equation used:

1 $$\frac{P/Po}{n(1-P/Po)}=\frac{1}{{n}_{m}c}+\frac{c-1}{{n}_{m}c}(P/Po)$$

where *P/Po* is the relative pressure, *n* is the amount of energy adsorbed per unit mass of the adsorbent, *n*_*m*_ and c are the adsorbent thermal parameters^22^.

### Hausdorff spectrum

It is a continuous spectrum of exponential values in bell-shaped appearance, that is used to describe a multifractal system. It evaluates the degree of nonlinearity in magnitude in the process generating the output *f*_*h*_(*α*). Multifractals are characterized by Holder exponent, defined as:

2 $$\alpha =\,\log \,\mu (box)/\,\log \,\varepsilon $$

where *µ*(box) is the box measure and *ɛ* is the dimension of the box^23^. The higher the value of *α*, the more regular is the signal. The study is based on the computation of the Holder regularity exponent at each point in the image. If *N*_*ɛ*_(*α*) is the number of boxes of sized *ε* for each value of *α*, then the Hausdorff dimension of the distribution of *α* is defined as:

3 $${{\rm{f}}}_{{\rm{h}}}(\alpha )=-\,\log \,{N}_{\varepsilon }(\alpha )/\,\log \,\varepsilon $$

### Legendre spectrum

It is a plot between Legendre fractal dimensions *f*_*l*_(*α*) and holder exponent *α*, and are obtained from XRD data in our case here. The Shape of the multifractal spectrum of singularities can be well captured by the width of the spectrum *Δα*_*l*_ which describes the range of probability and the difference of dimension *Δf*_*l*_, which quantifies the self-similarity in the fractals^23,24^. It is a very useful tool to describe and analyze the variations of nonlinear fractals of a system. Higher the value of *Δα*, higher is the probability of heterogeneity, which is the normal behavior of growth. Similarly, if the value of *Δf*_*l*_ < 0, it means that there are more concentrated regions than rarefied regions, indicating homogeneity at different geometrical scales and hence self-similarity in the samples considered.

### Lacunarity analysis

It is a tool used to analyze the surface properties in terms of the texture of various types of images of the object under study^25^. An algorithm was proposed by Allain and Cloitre that a box of size *r* slides over a whole image, such that, *S* is the number of pixels^26^. When the probability distribution *Q(S, r)* is obtained from the frequency distribution *n(S, r)* of the total number of box *N(r)*, then the Lacunarity of the given box size *r* is calculated as

4 $${\rm{L}}({\rm{r}})=\,\frac{{\rm{Z}}(2)}{{[{\rm{Z}}(1)]}^{2}}$$

where the moments *Z(1)* and *Z(2)* are calculated as:

5 $$Z(1)=\sum SQ(S,r)$$

6 $$Z(2)=\sum {S}^{2}Q(S,r)$$

Lacunarity also quantifies the degree of heterogeneity of a surface. The higher the variability of gaps indicated in a pattern, higher is the value of Lacunarity in the spatial pattern of the image under consideration, which predicts more heterogeneous texture^27^. Lacunarity analyses were used in measuring the distribution of cancer cells nuclei using digitized images. Lacunarity analyses have also been applied in medicine, image processing, geology, ecology and in many more fields^17^. In Materials Science, Lacunarity was used to determine the gaps in the nanostructure surface topography of molecular layers of retrograded starch in micro range in an extensive research work^25,26^. Hence, an attempt is made here for finding the lacunae distribution in the SEM images of the FZnO and BZnO samples for finding the surface properties.

### Photocatalytic experiment

The experiments are carried out in Methylene Blue (MB) dye solution^28,29^ in presence of sunlight. 100 mL (1 mg/L) MB dye solution is taken in a conical flask. To this, 0.05 mg of ZnO catalyst is added and stirred in dark for 10 min to allow the physical adsorption of dye molecules on the catalyst surface, followed by UV–vis absorption. The experimental setup is then placed at sunlight under constant stirring for an interval of 12 min followed by UV–vis absorption. The experiment is repeated several times with regular interval of time after exposure to sunlight^30^.

### Antibacterial assay

The concentrations of the samples made are 0.4 g/ml, 0.2 g/ml and 0.1 g/ml. The Luria Bertani (LB) Broth possesses nutrimental substance are taken in a 500 mL Erlenmeyer flask. The flask along with the laboratory glasswares were autoclaved at first at high pressure and temperature for sterilization. The test organisms, *E. coli* were lyophilized and procured in freeze-dried form. The uppermost of each colony of the pathogen was pick-up with a sterile loop and then moved to the Broth. The mixture was incubated at 37 °C (310 K) where cells were spread and attained a saturated phase after which turbidity corresponding to 0.5 McFarland densities was seen, indicated the fully grown organisms^11,16^. Here 0.5 McFarland’s standard was used during antibacterial susceptibility test and was carried out by well-diffusion method^31^. In this method, at first the sample ZnO were dissolved in de-ionized distilled water for making the certain required concentration. The initial concentration of solution was changed by diluting further to obtain various concentrations. The solutions are taken in micro centrifuge tubes and its vortex is carried out. Petri plates containing about 20 mL Muller-Hinton agar medium were left for 10 min to dry at the air and after that swabbed with culture of bacterial strains and wells of 6 mm in diameter were made for each concentration. The prepared solutions of different concentrations was added to each well and incubated for about 24 hrs. The antimicrobials present in the ZnO samples are allowed to diffuse out into the medium and interact in a plate freshly seeded with the test organisms. As the antimicrobial activity depends on the time of incubation after the samples are seeded into the petri plates, at the first hours, the zones were not visible but thereafter 24 hrs a clear zone of inhibition was seen around the wells of each plate. Triplicates were maintained and the experiments were repeated thrice, for each replicates the diameters of the zones were measured in three different fixed directions using vernier caliper and the average values were recorded.

## Results and Discussion

**Figure 1:**
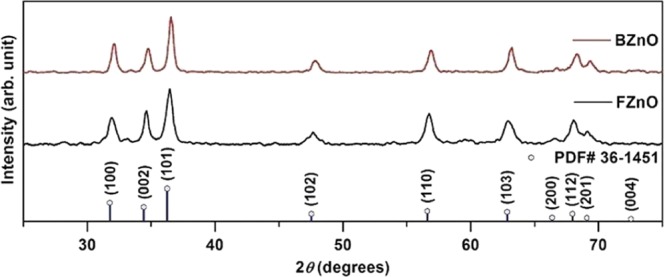
XRD profile of hexagon-shaped bulk ZnO and flower-shaped ZnO.

Figure 1 shows the X - ray diffraction pattern of the as-prepared samples. The peaks shown are well indexed *Powder Diffraction File* (PDF No. 36–1451), which indicates hexagonal phase of ZnO.

**Figure 2:**
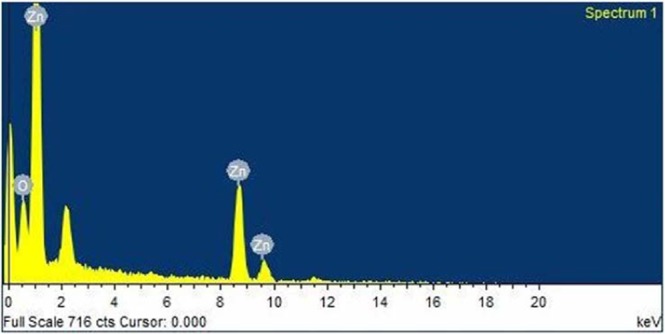
Energy Dispersive Analysis of X-rays spectrum of the sample FZnO.

The EDAX analysis shown in Fig. 2 indicates the presence of Zn and O for the ZnO samples and no other elements are found to be present in the samples^21,32^.

The EDAX spectrum shows well agreement with the experimental concentration used for the ZnO sample. Table 1 below gives the composition of elements in ZnO nanosample.

**Table 1:** Composition of elements in ZnO nanosample.

Element	Weight%	Atomic%
O K	14.16	40.26
Zn K	85.84	59.74
Total	100	100

The other sample BZnO is introduced for comparative study of the nonlinearity, to predict the strong possibility of FZnO in inhibiting microorganisms^5^. The values of optical band gap was obtained for BZnO and FZnO and were ~3.20 eV and 3.781 eV respectively as mentioned in the earlier paper^14^. Therefore, it may be concluded that the particles produced will possess optical properties, and hence they would show antibacterial activities for biological applications^11,14^. The average crystallite size of the ZnO NPs in the FZnO microstructure is found to be ~18.34 nm and that in case of the bulk is estimated as ~44.87 nm using de-bye Scherrer’s formula. It shows that particle size is quite small in the agglomeration of flower-shaped microstructure formed and indicates that when the grain size is small, it gives rise to thin layer formation and hence less complexity in the surface structure, manifested by the small width of the spectrum^33^.

**Figure 3:**
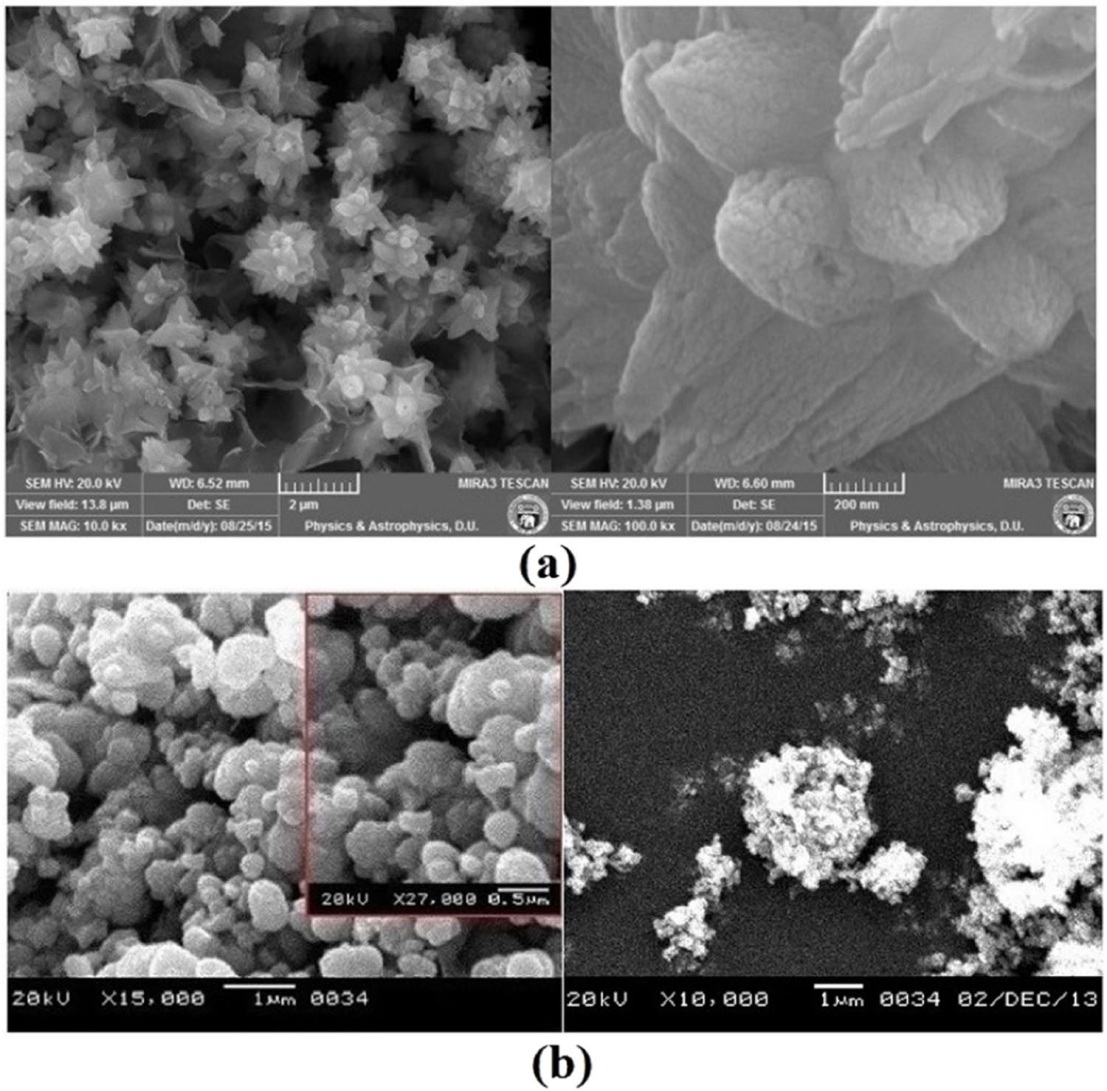
(**a**,**b**) FESEM image of small area of FZnO and BZnO microstructures.

Figure 3(a,b) show the FESEM image of the FZnO and BZnO respectively. It confirms the formation of FZnO having diameter of average size ~1.75–2.25 µm.

**Figure 4:**
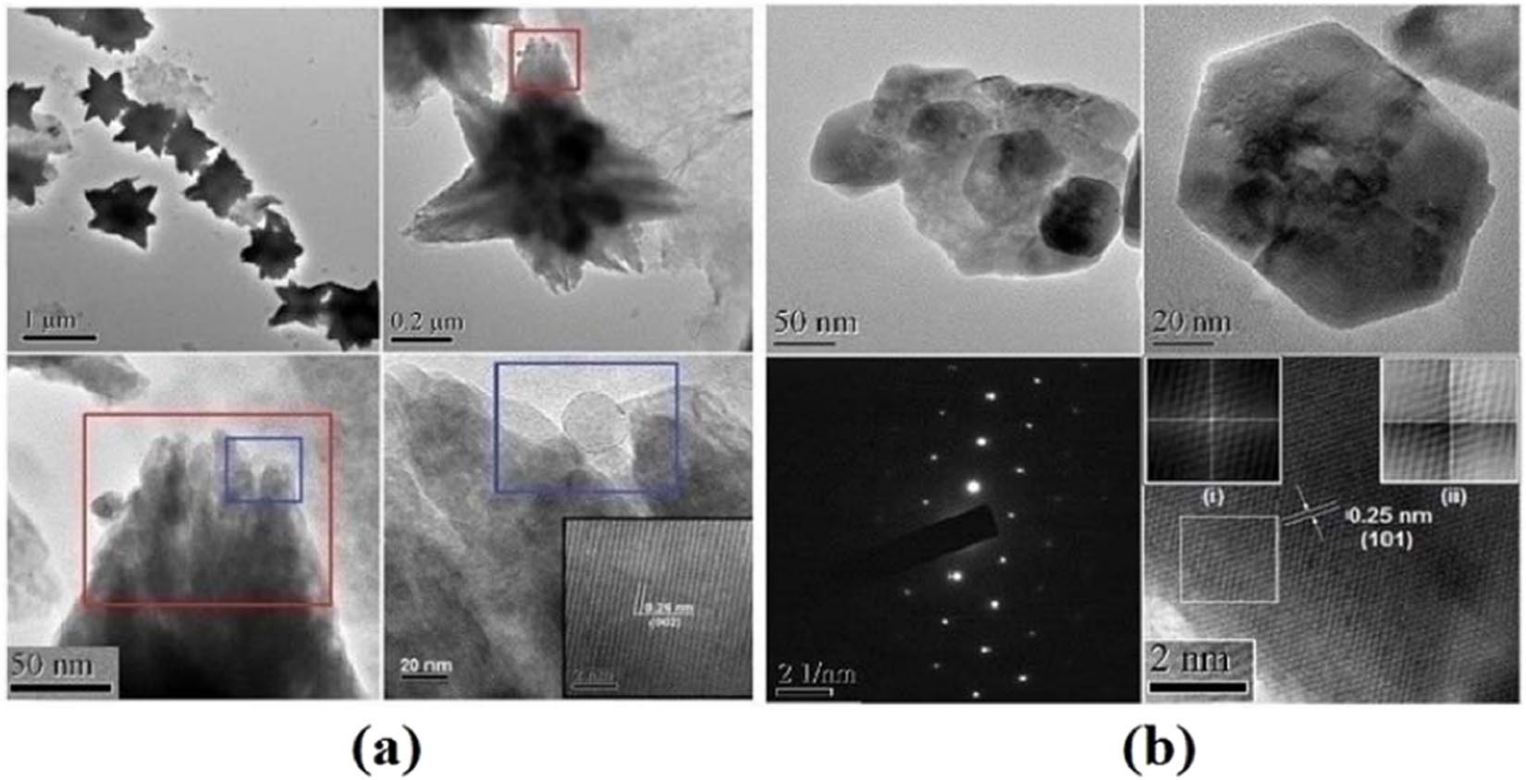
(**a**,**b**) HRTEM image of small area of FZnO and BZnO microstructures.

Figure 4(a,b) show the HRTEM image of the FZnO and BZnO microstructures respectively.

Figure 4(a) clearly shows the structure formation and also confirms relatively smaller granule sizes. It also verifies the phase structure and gives morphological details^16,34^ of the as-prepared samples. The length of each petal is found to be ~0.9–0.95 μm and all the petals have been oriented from a center point of the FZnO microstructure having diameter ~1.75 μm. Moreover, all petals are aggregation of ZnO NPs of an average particle size ~24.8 nm or slightly less than ~25 nm. The inter-planar spacing (*d*-spacing) value is measured to be ~0.26 nm, which corresponds to standard value for FZnO along (0 0 2) direction. Figure 4(b) shows HRTEM images of BZnO nanostructures (NSs) prepared without PVP. The micrograph reveals that particle sizes are in the range of ~36.71 nm to 51.80 nm. HRTEM image of hexagonal prismatic NSs is presented having an average particle size of ~47.92 nm. The inverse fast Fourier transform (IFFT) patterns of correlation and convolution lattice marked with a white square are seen clearly from insets [(i) & (ii)] of Fig. 4(b). The calculated *d*- spacing from HRTEM image of Fig. 4(b) is found to be ~0.25 nm, which matches closely with (1 0 1) plane of BZnO as expected, and selected area electron diffraction (SAED) pattern confirms that the BZnO NSs are crystalline in nature.

**Figure 5:**
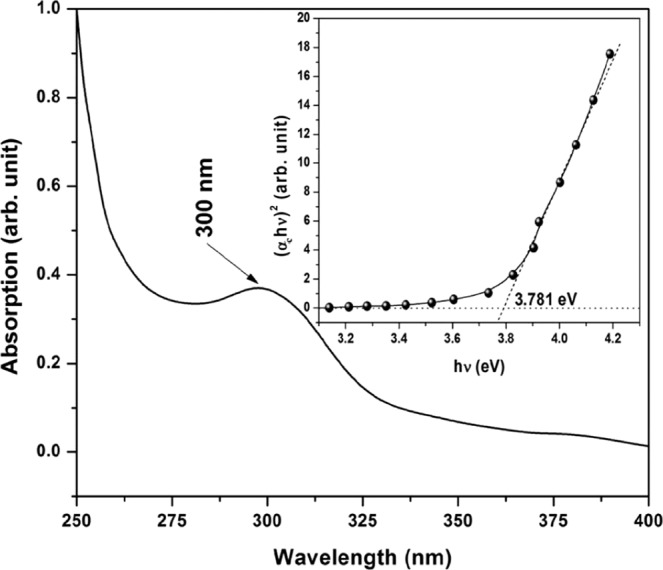
UV absorption spectra of FZnO.

The UV absorption spectra in Fig. 5 shows absorption peak at λ ~ 300 nm (4.13 eV), which indicates the presence of blueshift with respect to bulk ZnO (λ ~ 376 nm; 3.3 eV).

Using the plot of hν versus (α_c_ hν)^2^ for the absorption coefficient α_c_, as shown in the inset of Fig. 5, the direct energy gap of FZnO is calculated. The slope of the graph is related to the band gap *E*_*g*_ as (α_c_ hν)^2^ = k(hν−*E*_*g*_), where hν is the incident light energy and k is a constant^16,35,36^. The optical band gap value for FZnO is found to be nearly 3.781 eV and it is obtained by extrapolating the linear part of the graph.

**Figure 6:**
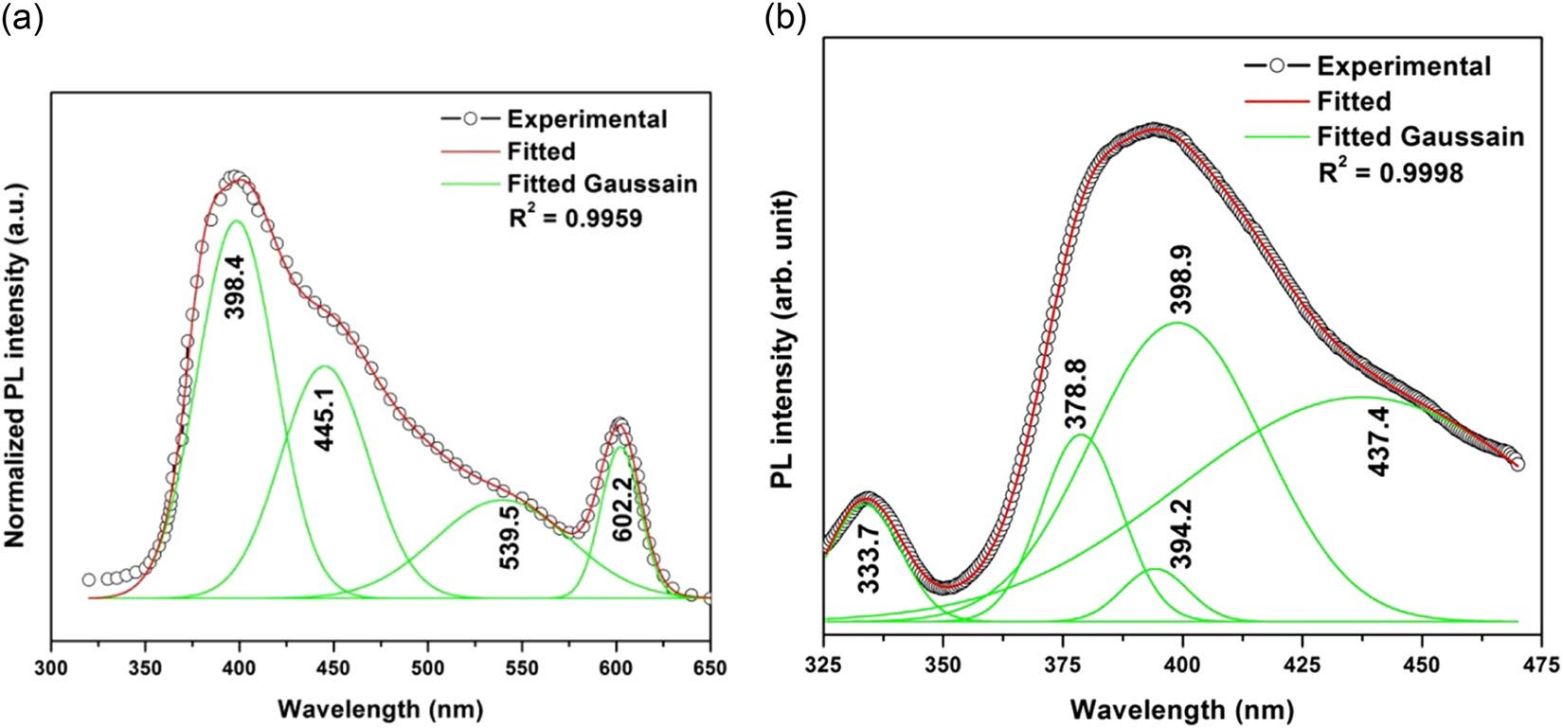
(**a**) Room temperature PL spectra of BZnO synthesized at 60 °C, using 0.1 M Zn(NO_3_)_2_.6H_2_O and drop-wise addition of NH_4_OH up to pH = 7.5 with addition of 3 wt% PVP (The Gaussian peaks marked as green colour at the bottom of the fitted curve marked with red colour). (**b)** Room temperature PL spectra of FZnO synthesized at 60 °C, using 0.075 M Zn(NO_3_)_2_.6H_2_O and drop-wise addition of NH_4_OH up to pH = 7.5 with addition of 3 wt% PVP (The Gaussian peaks marked as green colour at the bottom of the fitted curve marked with red colour).

Figure 6(a) shows the room temperature PL spectrum under excitation of 300 nm wavelength for sample BZnO and can be fitted by Gaussian peaks.

The position of peaks centers are estimated to be at ~398.4 nm (3.11 eV), ~445.1 nm (2.79 eV), ~539.5 nm (2.30 eV) and ~602.2 nm (2.06 eV). The ultra violet emission peak at ~398.4 nm associated with near-band-edge (NBE) emission^37^, while blue emission peak at ~445.1 nm indicates the emission due to Zn interstitials. The green PL peak appeared at ~539.5 nm in the PVP capped sample is associated with oxygen vacancy and yellow emission peak at ~602.2 nm is due to oxygen interstitials.

Figure 6(b) shows the room temperature PL spectrum of FZnO NSs under excitation wavelength of 300 nm and it is obtained by deconvoluting the peaks using Gaussain functions^22^.

The position of the peaks obtained are at XC1 = 333.7 nm (3.72 eV), XC2 = 378.8 nm (3.28 eV), XC3 = 394.2 nm (3.15 eV), XC4 = 398.9 nm (3.11 eV) and XC5 = 437.4 nm (2.83 eV). The UV emission peaks (XC1, XC2, XC3 and XC4) seen in the FZnO corresponds to its near band-edge (NBE) emission, which are due the recombination of free excitons^38,39^. The peak that is centered at XC1 attributes to the energy gap of the ZnO NPs, which is the blue-shift on account of its quantum confinement effect. In the same manner, the peak that is centered at XC2 is linked to the band edge transition from conduction band (CB) to valence band (VB). Similarly, the peak that is centered at XC4 corresponds to the transition related to Zn vacancy^40^ and the weak emission peak that is centered at XC5 corresponds to blue emission and is associated with Zn interstitial defects^41^.

**Figure 7:**
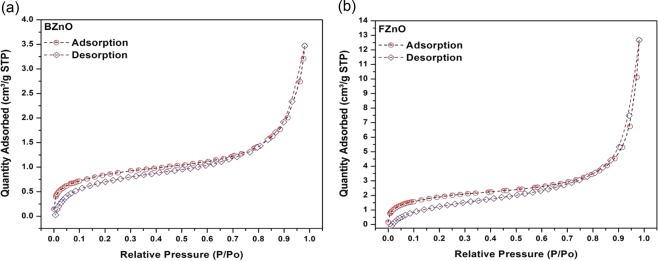
(**a)** Nitrogen adsorption–desorption isotherms for the material of BZnO sample. (**b)** Nitrogen adsorption–desorption isotherms for the material of FZnO sample.

If the value of energy from PL peak position XC2 = 378.8 nm (3.28 eV) is added with the free exciton binding energy value of bulk ZnO (60 meV), then approximately similar value of the FZnO band gap energy at room temperature will be obtained. Therefore, it can be said that the sample FZnO as-prepared indicates high quality in nature, since free exciton luminescence is observed only in pure material with a high structural quality. Figure 7(a,b) shows the nitrogen adsorption-desorption isotherms for the material of BZnO and FZnO samples respectively.

The plot for FZnO is steeper than that for BZnO as seen in Fig. 7. It indicates that the rate of adsorption of adsorbate N_2_ gas by the adsorbent FZnO is higher than that of BZnO, which is due to the complex surface structures, formed as a result of condensation of minute particles into the mesoparticles during the synthesis process. In turn, this resulted in formation of rough surface of the FZnO, which is also suggested by the NLD analysis discussed later. This lead to increased activation of the adsorbent. The sharp rise in the adsorbed quantity in the vicinity of 0.9 relative pressure, is due to inter-particle condensation into domains^32^, which is much higher for FZnO. This may be because of the fact that such granules adsorb substantial adsorbate due to their smaller particle size and larger effective area (see Table 2) as a result of agglomeration into microstructure.

**Table 2:** Textural Parameters of the ZnO materials obtained from nitrogen adsorption isotherms.

Sample	BET Surface Area (m^2^/g)	BJH Adsorption Cumulative Surface Area of Pores (m^2^/g)	Micropore Surface Area (m^2^/g)	Adsorption Average Pore Width (nm)
BZnO	2.9266	1.6843	2.8119	7.3344
FZnO	6.7143	3.9595	7.8782	11.6783

**Figure 8:**
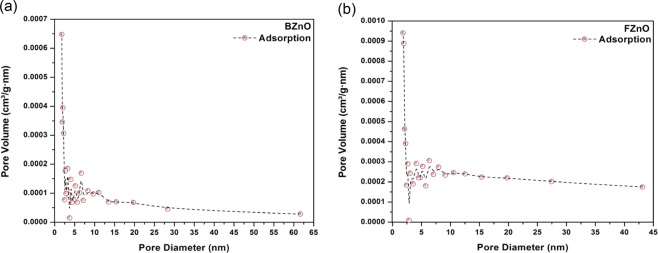
(**a)** BJH Pore volume vs. pore diameter (nm) for BZnO materials determined from the nitrogen adsorption of the particles formed. (**b)** BJH Pore volume vs. pore diameter (nm) for FZnO materials determined from the nitrogen adsorption of the particles formed.

Figure 8(a,b) illustrate the dependence of the pore size distribution with the BJH pore volume and the pore diameter for BZnO and FZnO respectively.

The pore size distribution of both the samples show the dependence of the BJH pore volume on the pore diameter^32^. The narrow pore size distribution for FZnO and its greater magnitude, compared to that of BZnO, shows the difference in their specific surface areas (see Table 2).

The higher specific pore volume within narrow pore size distribution for FZnO confirms its richer surface texture and higher porosity, thereby giving rise to higher exposure of contact surface, indicating possibility of its pronounced microbial inhibition, as suggested by multi fractal analysis too. The textural parameters of ZnO materials of both BZnO and FZnO obtained from nitrogen adsorption isotherms at 77 K are shown in Table 2.

It is found that the average pore size of BZnO is lesser than that of FZnO, which indicates that the surface-to-volume ratio of the granules of BZnO is smaller than that of FZnO. Thus, the inhibitory activity of FZnO for given micro-organisms can be expected to be more than that of BZnO^42^. This fact is also seen from the BJH analysis of the samples (see Fig. 8).

**Figure 9:**
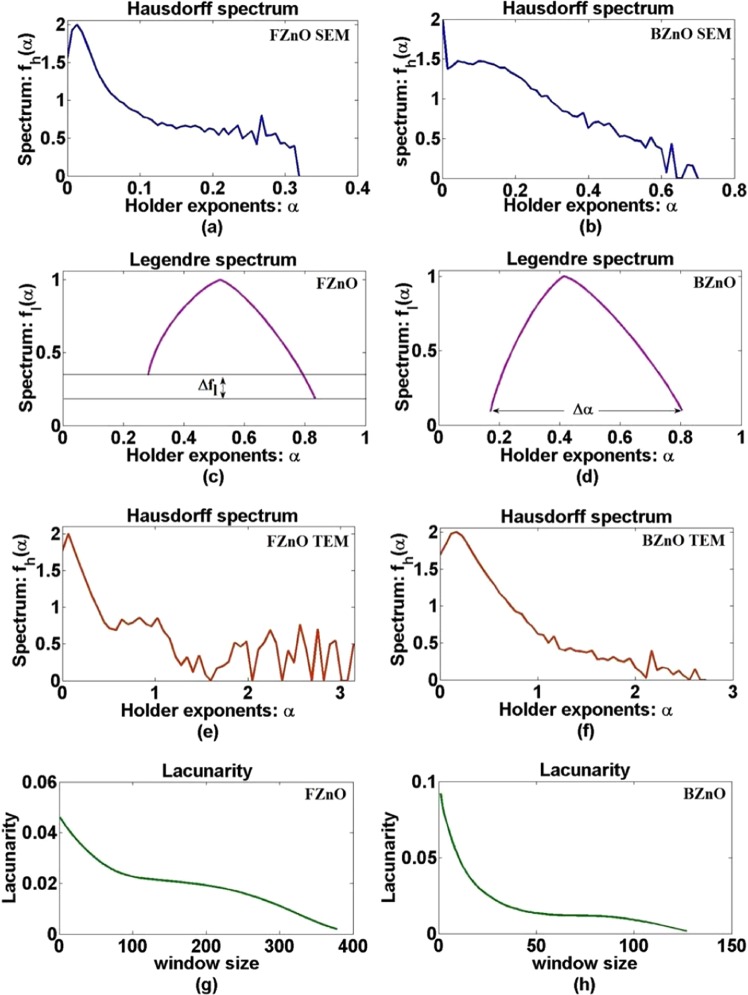
(**a**,**b**) Hausdorff spectrum of FESEM image of flower-shaped and hexagon-shaped bulk ZnO, (**c**,**d**) Legendre spectrum of flower-shaped and hexagon-shaped bulk ZnO. (**e,f**) Hausdorff spectrum of HRTEM image of flower-shaped and hexagon-shaped bulk ZnO. (**g**,**h**) Lacunarity spectrum of flower-shaped and hexagon-shaped bulk ZnO.

Figure 9(a,b) show respective Hausdorff Spectra of the SEM images.

It follows that the width (*Δα*) is smaller in FZnO than that in BZnO, indicating that the texture of FZnO to be smoother and more geometrical with richer patterns in comparison to that of BZnO^23^, resulting in a lower width of the singularity curve, confirming self-similarity at different scales (see Fig. 9). The above results show the richness in the surface structures of FZnO in comparison to that of BZnO. Hence it may be predicted that the FZnO would be a better inhibitor.

Figure 9(c,d) show Legendre Spectrum of the XRD of the FZnO and its BZnO samples respectively. A relatively smaller value of *Δα* and higher value of *Δf*_*l*_ in case of FZnO predicts rich pattern formation^23^. Figure 9(e,f) show respective Hausdorff spectrum of the HRTEM images. They show similar plots as Fig. 9(a,b) and hence confirm the same inferences.

Figure 9(g,h) show Lacunarity spectrum of the FESEM images of the FZnO and BZnO respectively. The value of Lacunarity for FZnO and BZnO are measured at different window sizes. The Lacunarity measured at different scales for FZnO are larger than that for BZnO. The larger value in case of FZnO indicates that the gaps in the surface structure are larger and hence patterns are richer at the surface, thereby qualifying itself to be a better inhibitor of microorganisms and, may be, a prospective candidate for other biological applications we propose to take up in future.

**Figure 10:**
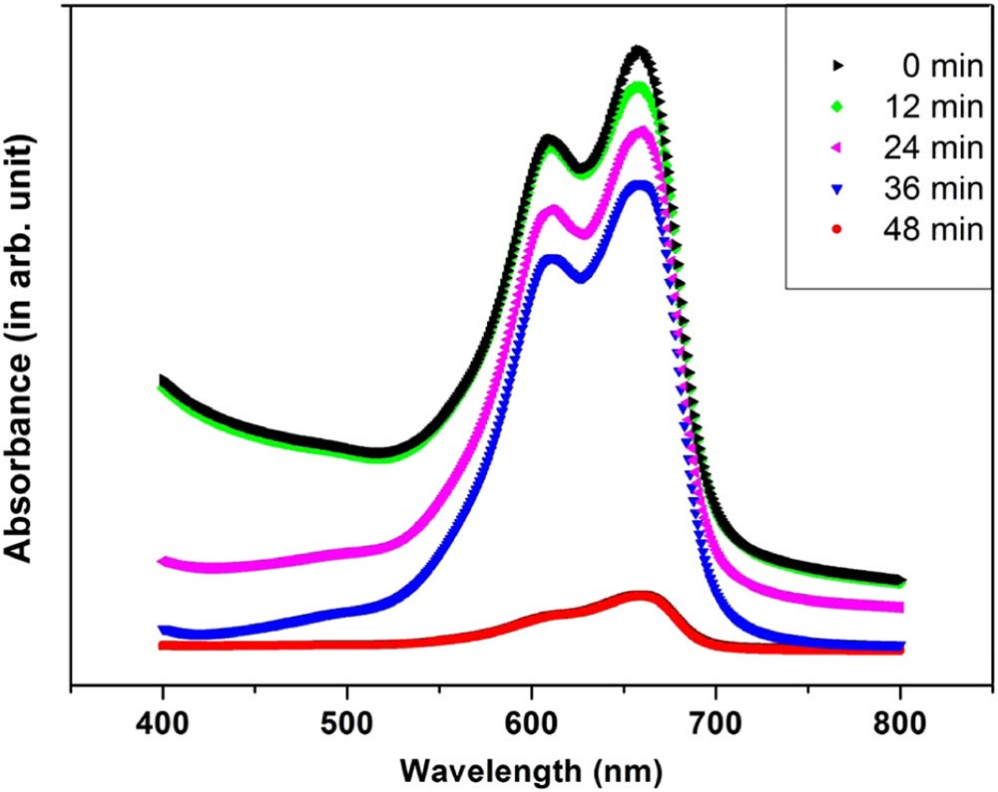
Photocatalytic degradation of Methylene blue by the FZnO under sunlight irradiation.

Figure 10 shows the Photocatalytic degradation of Methylene blue by the FZnO under sunlight irradiation. The peaks of the graphs decrease with increase in time exposure to sunlight, confirming strong photocatalytic properties. The fall in peak or degrade of dye concentration is seen to be faster between 36 and 48 minutes. It is because of fact that the total effective exposure time is maximum when it reaches 48 minutes, thereby causing the concentration of the dye go down abruptly, due to efficient photo-generated electron-trapping, ensuring the suppression of electron-hole recombination. Further, it was also reported that a good photocatalytic material is a good inhibitor of bacteria too. Nanoparticles accelerate the creation of e^−^– h^+^ pairs in them and photo-generated e^−^ recombines with O_2_ which is a partial process of photocatalytic activity^42,43^. Also the photocatalytic reduction activity of ZnO gets improved, due to fast gaining of electrons from the water present in the solution^44^. It is reported in SWang et al. (2018). O microstructures shows enhanced photocatalytic degradation activity^45^. Therefore, FZnO should find major biological applications, and for this, antibacterial susceptibility test^11^ is carried out.

**Figure 11:**
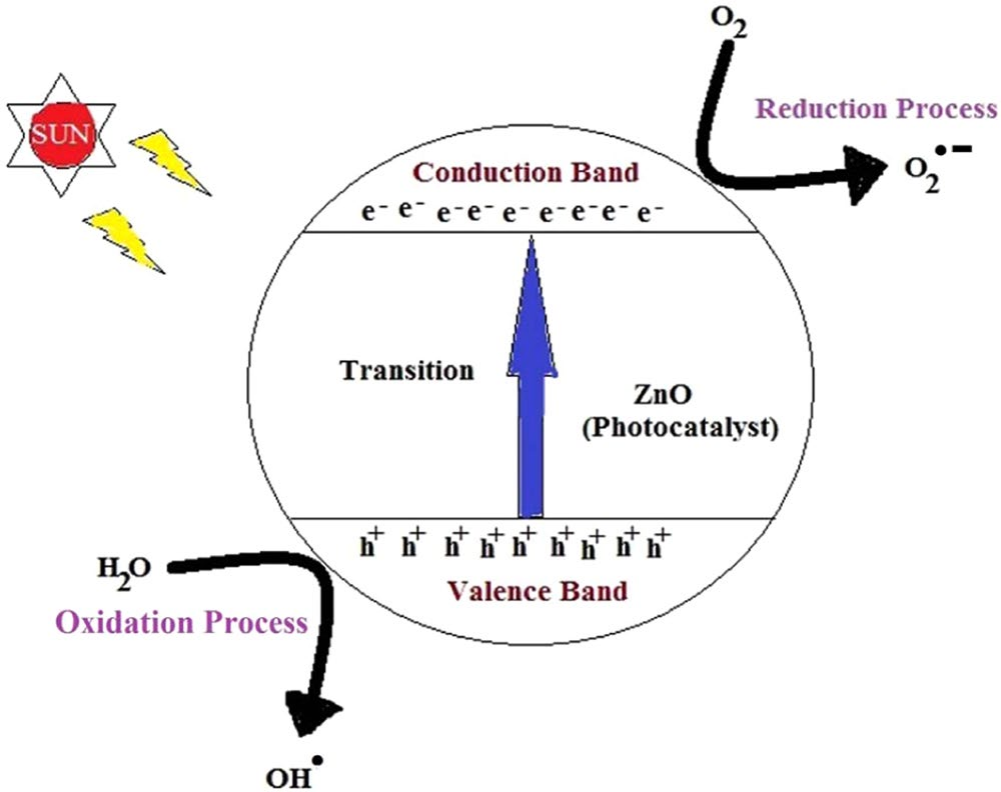
Photodegradation mechanism of MB dye using ZnO as catalyst.

In general, photocatalytic activity takes place in four steps. It begins with creation of electron–hole (e^−^– h^+^) pairs when light is incident to the photocatalyst, followed by separation and migration of charge carriers to the surface, thereafter oxidation and finally reduction takes place on the surface of the catalyst^46,47^. As Sun light has energies greater than the energy gap of ZnO, transition of charge from valence to conduction band takes place, thereby creating the e^−^– h^+^ pairs. Subsequently, the photo-generated electrons accumulated on the surface of the catalyst get combined with the O_2_ adsorbed in it, and as a result superoxide anion radicals (${{\rm{O}}}_{2}^{\cdot -}$) are produced. At the same time, the oxidized atoms in the surface of ZnO reacts with water to form ${{\rm{OH}}}^{\cdot }$ (see Fig. 11). Larger the exposure time during the experiment, higher is the number of ${{\rm{OH}}}^{\cdot }$ formed. Similarly, richer the surface structure of the photocatalyst, better is the activity^36,48^. These radicals ${{\rm{O}}}_{2}^{\cdot -}$ and ${{\rm{OH}}}^{\cdot }$ decompose the MB and organic contaminants into CO_2_ and H_2_O as depicted below^21,49^:

$${\rm{ZnO}}+{\rm{h}}\upsilon \to {{\rm{e}}}^{-}+{{\rm{h}}}^{+}$$

$${{\rm{O}}}_{2}+{{\rm{e}}}^{-}\to {{\rm{O}}}_{2}^{\cdot -}$$

$${{\rm{H}}}_{2}{\rm{O}}\to {{\rm{OH}}}^{-}+{{\rm{H}}}^{+}$$

$${{\rm{OH}}}^{-}+{{\rm{h}}}^{+}\to {{\rm{OH}}}^{\cdot }$$

$${{\rm{OH}}}^{\cdot }/{{\rm{O}}}_{2}^{\cdot -}+{\rm{MB}}\to {\rm{degradation}}\,{\rm{products}}+{{\rm{CO}}}_{2}+{{\rm{H}}}_{2}{\rm{O}}$$

**Figure 12:**
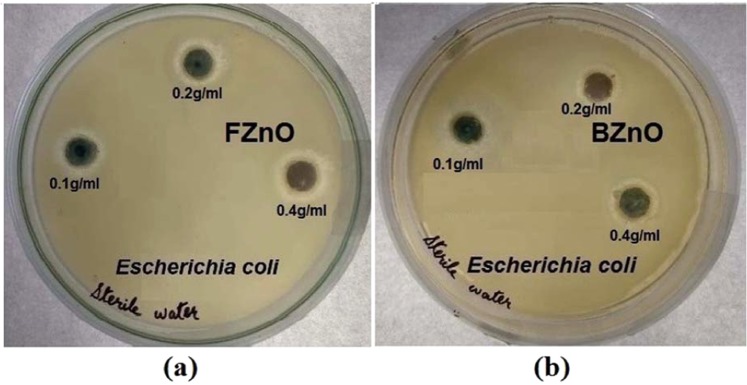
(**a**,**b**)Antibacterial activity of flower-shaped ZnO and hexagon-shaped bulk ZnO.

Figure 12(a,b) show the Zones of Inhibition (ZoI) of the gram-negative microorganisms inhibited by the as-prepared FZnO sample and BZnO sample respectively.

The values of ZoI at the concentration of 0.4 g/ml of FZnO and BZnO for *E. coli* pathogens are found to be 2.5 cm and 2 cm respectively. It is seen that the zones are larger in Fig. 12(a) than Fig. 12(b), which validates our prediction reached at from the nonlinear dynamical analysis reported above that the FZnO particles are better inhibitors of *E. coli* than the BZnO. It is because, area of zone of inhibition depends on the particle size as well as in the particle shape of the sample. The calculated average granule size of BZnO is nearly 44.87 nm and that of flower-like ZnO microstructure is nearly 18.34 nm. Also, FZnO has a complex surface structure whose number of facets are very much larger than that of BZnO as proved by the MFA analysis, enhancing its antimicrobial activities by increasing the effective contact area with the pathogens under test. The increase in the number of facets too shows higher degree in fractality and therefore the Lacunarity analyses are made for the samples for predicting their difference in inhibitory capabilities. The complex structure, increases surface area and thereby the adsorptions of bacterial proteins are increased, which in turn increases bacterial inhibition^50^.

**Figure 13:**
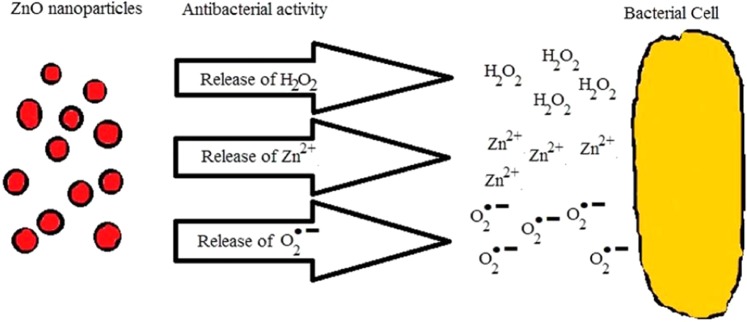
Antibacterial mechanism of ZnO.

The formation of photo-generated e^−^– h^+^ pairs, and hence the formation of radicals in photocatalytic experiment, are some of the factors are responsible for the antibacterial effect of ZnO particles as suggested by several authors^21,51^. Thereby, the photocatalytic activity of FZnO is carried out as the first step for the analysis. Some of the probable manners which lead to bacterial cell death due to ZnO NPs are discussed in brief here. Firstly, it is believed that the ZnO NPs might have released the Zn^2+^ when in contact with the bacterial cell membrane, allowing the latter penetrate through the cell and react with the content inside it^51,52^. Secondly, they are capable of generating some oxidizing agents, such as, hydrogen peroxide (H_2_O_2_), which are good enough in damaging the cell wall of bacteria. Lastly, they might have generated superoxide radicals ${{\rm{O}}}_{2}^{\cdot -}$ which attack the cellular content and kill it^53^ (see Fig. 13).

The mechanism for the production of the above mentioned reactive oxygen species (ROS) in ZnO nanoparticles are shown as^54,55^:

$${\rm{ZnO}}+{\rm{h}}\upsilon \to {{\rm{e}}}^{-}+{{\rm{h}}}^{+}$$

$${{\rm{O}}}_{2}+{{\rm{e}}}^{-}\to {{\rm{O}}}_{2}^{\cdot -}$$

$${{\rm{H}}}_{2}{\rm{O}}+{{\rm{h}}}^{+}\to {{\rm{OH}}}^{-}+{{\rm{H}}}^{+}$$

$${{\rm{O}}}_{2}^{\cdot -}+{{\rm{H}}}^{+}\to {{\rm{HO}}}_{2}^{\cdot }$$

$${{\rm{HO}}}_{2}^{\cdot }+{{\rm{H}}}^{+}+{e}^{-}\to {{\rm{H}}}_{2}{{\rm{O}}}_{2}$$

$${\rm{ZnO}}+{{\rm{H}}}_{2}{\rm{O}}+Energy\to {{\rm{ZnO}}}^{\ast }\to {{\rm{Zn}}}^{2+}+{{\rm{H}}}_{2}{{\rm{O}}}_{2}$$

The rate of formation of Zn^2+^ can be enhanced by increasing the concentration of ZnO^52^.

## Conclusion

Using wet chemical method, FZnO and BZnO microstructures are successfully synthesized at 60 °C with de-ionized (DI) water as a solvent. From the analysis of the XRD obtained, the hexagonal phases of ZnO as well as its inherent nonlinearity are observed. The SEM and TEM images confirm FZnO with diameter ~1.75–2.25 µm. The smaller width (*Δα*) and high value of *Δf*_*l*_ in FZnO, indicates its texture to be smoother and more geometrical with richer patterns in comparison to that of BZnO, which is substentiated by the BET surface area analysis. Hence it may be predicted that the FZnO would be a better inhibitor. The calculation of Lacunarity of FZnO is found to be larger in different scales than that of BZnO, also confirmed from the porosity distribution of the particles formed, indicating richness in the surface structure, thereby predicting it to be a better inhibitor. The photocatalytic activity of FZnO structure proves that it is a good photocatalyst and, therefore, finds application in biological applications, as predicted by the nonlinear dynamical analysis. The antibacterial activities of the FZnO with organism *E. coli* shows pronounced inhibition. Thus, it is confirmed that Lacunarity is an efficient tool to identify materials with rich surface structure or patterns formed.
